# Association of adenotonsillar disease and adenotonsillectomy with the development of vitiligo: A nationwide population-based cohort study

**DOI:** 10.3389/fmed.2022.1004218

**Published:** 2022-11-03

**Authors:** Jong Seung Kim, Min Gyu Lee, Sang-Kyung Lee, Sang-Woo Yeom, Min-Gu Kang, Jong Hwan Lee, Il-Jae Lee, Jin Park, Seok-kweon Yun, Kyung-Hwa Nam

**Affiliations:** ^1^Department of Otorhinolaryngology, Head and Neck Surgery, Jeonbuk National University Medical School, Jeonju, South Korea; ^2^Department of Medical Informatics, Jeonbuk National University, Jeonju, South Korea; ^3^Research Institute of Clinical Medicine of Jeonbuk National University – Biomedical Research Institute, Jeonbuk National University, Jeonju, South Korea; ^4^Department of Dermatology, Jeonbuk National University Medical School, Jeonju, South Korea

**Keywords:** adenoids, tonsil, adenoidectomy, tonsillectomy, vitiligo

## Abstract

**Background:**

Vitiligo is a common acquired skin depigmentation disorder and is associated with various other autoimmune diseases which include thyroid disease and rheumatoid arthritis. Similarly, adenotonsillar disease (ATD) may induce inflammatory or autoimmune diseases in other organs which include the skin. However, the influence of ATD on the development of vitiligo has not been studied.

**Objectives:**

To determine the association between ATD and adenotonsillectomy, and the development of vitiligo.

**Design and methods:**

Using data from the National Health Insurance Service database, patients diagnosed with ATD between 2008 and 2010 were included in the study. We performed two rounds of 1:1 propensity score matching in the ATD and adenotonsillectomy groups. The ATD and non-ATD groups both included 206,514 individuals. Among the ATD group, the adenotonsillectomy and non-adenotonsillectomy groups both included 23,354 individuals. Each individual was monitored until 2019. The primary end point was the risk of vitiligo. Using the Cox Proportional Hazards model, the incidence of vitiligo and the hazard ratio (HR) were calculated.

**Results:**

The incidence of vitiligo was 1.16-fold higher in the ATD group than in the non-ATD group [adjusted HR (aHR), 1.16; 95% confidence interval (CI), 1.09–1.24] and 0.82-fold lower in the adenotonsillectomy group than in the non-adenotonsillectomy group (aHR, 0.82; 95% CI, 0.68–0.99). Additionally, the other risk factors for developing vitiligo included thyroid disease (aHR, 1.48; 95% CI, 1.11–1.98), age younger than 30 years (aHR, 1.18; 95% CI, 1.09–1.27), and age over 60 years (aHR, 1.22; 95% CI, 1.06–1.41), whereas factors including rural residency (aHR, 0.91; 95% CI, 0.85–0.98) and low economic status (aHR 0.87; 95% CI, 0.82–0.93) were associated with decreased incidence of vitiligo.

**Conclusion:**

In this study, ATD increases the risk of vitiligo and adenotonsillectomy attenuates its development. Clinicians should consider ATD as a pathogenic factor for vitiligo and the potential effect of adenotonsillectomy in its management.

## Introduction

Vitiligo is a common acquired depigmentation disorder of the skin arising from selective destruction of melanocytes and has an estimated prevalence of 0.5–1% in the global population (1). The prevalence differs by country, with India reporting the highest at 8.8%, followed by Mexico (4%), Japan (1.68%), Denmark (0.38%), and Korea (0.12–0.13%) (2–6). Although the exact etiology of vitiligo is not fully understood, it is multifactorial, and autoimmunity is considered to be the principal contributor to its pathogenesis (1). CD8 + T-cells are known to play a major role in its development. In addition, the association of vitiligo with a variety of other autoimmune diseases, such as thyroid diseases, rheumatoid arthritis, psoriasis, Addison’s disease, adult-onset diabetes mellitus (DM), alopecia areata, pernicious anemia, and systemic lupus erythematosus, has been reported (1, 7).

Tonsils are a pair of lymphoid tissues located at the rear of the throat that function as the first line of defense against bacteria and viruses. The nasopharynx-associated lymphoid tissue (NALT) is an antigen-specific immune response-inducing tissue mainly composed of the Waldeyer’s ring comprising the tongue, ear canal, plate, and nasopharyngeal tonsil (adenoid), and acts as the first barrier against foreign antigens (8, 9). The concept of “tonsillar focal disease” has been used for a long time, and is described as “a disease that induces responsive organic or functional injury to other organs distant from the tonsils, with the tonsils as the focus” (10). Tonsillectomy has been reported to be the effective primary treatment for a variety of tonsillar focal diseases which include palmoplantar pustulosis, pustulotic arthro-osteitis, and IgA nephropathy; moreover, there are various other diseases for which tonsillectomy has proved to be effective (10, 11). These diseases are collectively referred to as tonsil-induced autoimmune/inflammatory syndrome (TIAS) (11). Therefore, it would be helpful to study the association between the development of vitiligo and adenotonsillar disease (ATD) which may cause inflammatory or autoimmune diseases in other organs which include the skin, and which has not been studied yet.

On the other hand, a Swedish cohort study has suggested that the incidence of autoimmune diseases is increased in individuals who underwent adenotonsillectomy (12). In this study, a variety of rheumatologic disorders, type 1 DM, Graves’ disease, Hashimoto thyroiditis, and psoriasis were included, but vitiligo was not. Additionally, a Korean birth cohort study showed that undergoing adenotonsillectomy in childhood might be associated with an increased risk of alopecia areata, while the association of adenotonsillectomy with the risk of psoriasis and vitiligo was statistically non-significant (13). Although that Korean cohort study was the first to evaluate the association between adenotonsillectomy and vitiligo, ATD was not considered an antecedent factor for autoimmune diseases and those authors only studied patients under the age of 9 years who underwent adenotonsillectomy.

In this study, we aimed to investigate the risk of vitiligo development in patients diagnosed with ATD, and the effect of adenotonsillectomy on vitiligo development using the Korean National Health Insurance Service (NHIS) claims database which includes individuals of all ages.

## Materials and methods

### Data source

The Korean NHIS, mandated by law, covers up to 98% of the 50 million population of South Korea, and the NHIS database has been used to provide reliable estimates of the prevalence of certain diseases in the country (14). We used the NHIS-National Sample Cohort (NHIS-NSC) database which contains sample data from 3.5 million individuals, which is 7% of the total population of Korea and the data were randomly stratified (NHIS 2021-1-689). These data included general medical history, such as diagnosis code, admission date, medical practice code, age, sex, region of residence, economic status, and information on mortality.

### Study populations

**FIGURE 1:**
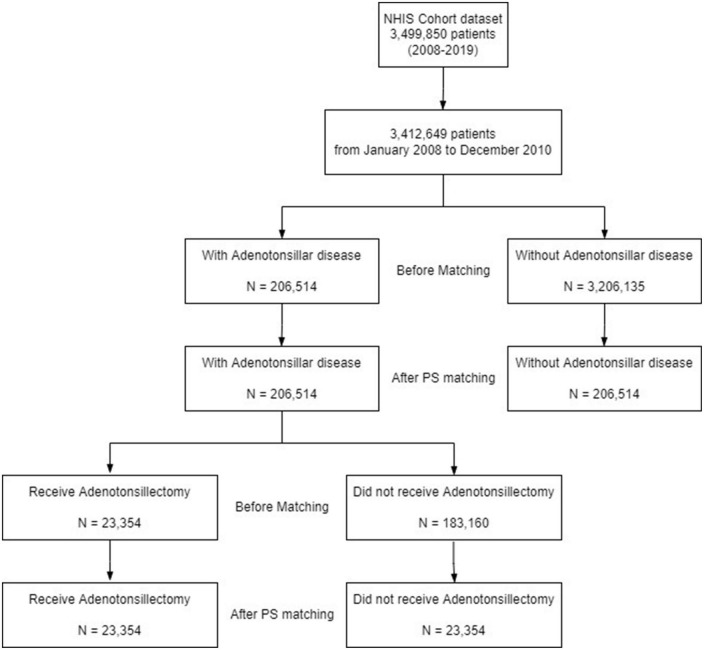
Study flow chart. NHIS, National Health Insurance Service; PS, propensity score.

Figure 1 shows the study design.

#### Study group

The study group was defined as the ATD group and included individuals diagnosed with International Classification of Diseases, 10th revision (ICD-10) code J35 at least once. Study outcomes are based on ICD-10 codes, which is a validated approach of diagnosis (Supplementary Table 1) (15–17). The inclusion criterion was patients diagnosed with adenoid and/or tonsillar disease between 2008 and 2010. The exclusion criteria were as follows: (1) patients with a diagnosis of ATD between 2011 and 2019, and (2) patients diagnosed with vitiligo before ATD diagnosis. A total of 206,514 patients were enrolled in the ATD group.

#### Control group

The control (non-ATD) group was composed of patients who were not diagnosed with adenoid and/or tonsillar disease. To include only patients with a propensity similar to that of the ATD group and to avoid potential bias from confounders, the non-ATD group was obtained by 1:1 propensity score (PS)-matching with the ATD group considering age, sex, economic status, residential area, and underlying diseases which included hypertension (HTN), DM, chronic kidney disease (CKD), thyroid disease which includes Graves’ disease and Hashimoto’s thyroiditis (GD/HT), and rheumatoid arthritis (Supplementary Table 1). A total of 206,514 patients were enrolled in the non-ATD group.

#### Subgroup analysis based on adenotonsillectomy status

Of the 206,514 patients in the ATD group, 23,354 underwent adenoidectomy and/or tonsillectomy, which was defined as the adenotonsillectomy group (Supplementary Table 1). Accordingly, a non-adenotonsillectomy group was obtained by 1:1 PS-matching with the adenotonsillectomy group, considering age, sex, economic status, residential area, HTN, DM, CKD, GD/HT, and rheumatoid arthritis. Finally, the adenotonsillectomy and non-adenotonsillectomy groups both enrolled 23,354 individuals. Any major imbalance between the two groups was identified quantitatively by standardized mean difference (SMD).

### Analysis conditions

For age, we divided each group into three subgroups (< 30, 30–59, and ≥ 60 years). Economic status was subdivided by income quantile; the top 30% was defined as high economic status and the bottom 70% as low economic status. Three levels were set for residential areas (Seoul, Metropolitan, and Rural). For underlying diseases, we evaluated HTN, DM, CKD, GD/HT, and rheumatoid arthritis.

### Major outcome and analytical methods

The major outcome was the occurrence of vitiligo. A diagnosis of vitiligo was defined as a patient with at least one documented physician contact with a principal diagnosis of ICD-10 code L80 during the observation period. Each individual was followed up until 31 December, 2019. Using the Cox Proportional Hazards model, the hazard ratio (HR) and 95% confidence interval (CI) were used to evaluate the association between ATD, and demographic factors, underlying diseases, comorbid diseases, and the risk of vitiligo. In subgroup analysis, the HR for vitiligo in relation to adenotonsillectomy was also calculated. All analyses were performed using SAS statistical software (version 9.4; SAS Institute, Cary, NC, USA) and R 4.0.3 statistical program (R Foundation for Statistical Computing, Vienna, Austria).

### Sensitivity analyses

Significant heterogeneity may be present in our data since unobserved covariates may be present but not included in our study’s multivariate Cox proportional hazard model. In addition, the Cox proportional hazards model assumes that the survival times of each patient are independent of each other. For this reason, a Cox frailty model was also performed to account for possible correlations between PS-matched pairs. In addition to the Cox frailty model, five sensitivity analyses were also performed to harden the robustness of our results. These showed that the results did not change based on the following: (1) operational definitions for vitiligo (increasing the number of vitiligo diagnoses from “1 or more” to “4 or more”); (2) statistical methods [Cox frailty model, PS matching with replacement, Inverse Probability of Treatment Weighting (IPTW), greedy nearest neighbor]; (3) matching variables: before matching, minimal matching (age and sex), demographic matching (age, sex, residential area, economic status), and full matching (age, sex, residential area, economic status, all underlying diseases); (4) recruitment periods for the ATD group (5, 4, and 3 years); (5) matching ratios (1:4, 1:3, 1:2, 1:1).

## Results

### Validation of the propensity score-matching

Table 1 shows SMDs between the ATD and non-ATD groups after PS-matching. None of the SMDs exceeded 0.05, which indicates that, among the nine independent variables, there were no statistically significant differences between the two groups. Additionally, in subgroup analysis, there were no significant imbalances between the adenotonsillectomy and non-adenotonsillectomy groups (all SMD < 0.05) (Supplementary Table 2).

**TABLE 1:** Characteristics of the study (ATD) and control (non-ATD) populations.

Variable	Study group (*n* = 206,514)	Control group (*n* = 206,514)	SMD
**Sex**			0.00
Male	97,814	98,108	
Female	108,700	108,406	
**Age**			0.01
< 30	129,133	129,800	
30–59	64,500	64,103	
≥ 60	12,881	12,611	
**ES**			0.00
Low	135,394	135,354	
High	71,120	71,160	
**Region**			0.00
Rural	101,145	101,035	
Metro	60,182	60,227	
Seoul	45,187	45,252	
**HTN**			0.01
Yes	17,480	17,096	
No	189,034	189,418	
**DM**			0.02
Yes	8,863	8,012	
No	197,651	198,502	
**CKD**			0.02
Yes	5,949	5,152	
No	200,565	201,362	
**GD/HT**			0.02
Yes	1,827	1,442	
No	204,687	205,072	
**RA**			0.02
Yes	6,367	5,580	
No	200,147	200,934	

ATD, adenotonsillar disease; ES, economic status; HTN, hypertension; DM, diabetes mellitus; CKD, chronic kidney disease; GD, Graves’ disease; HT, Hashimoto’s thyroiditis; RA, rheumatoid arthritis; SMD, standardized mean difference.

### Incidence of vitiligo in the adenotonsillar disease and adenotonsillectomy groups

**FIGURE 2:**
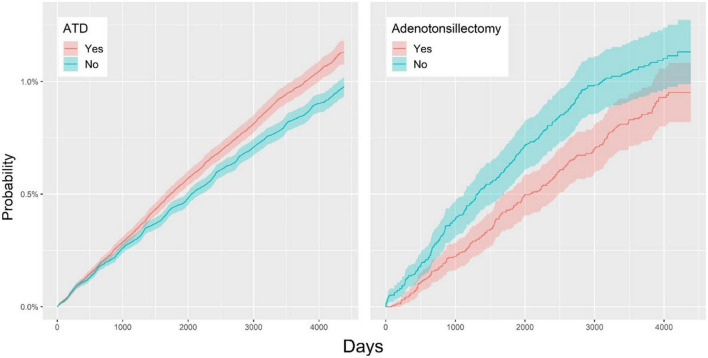
Overall cumulative incidence rates for vitiligo in the ATD and non-ATD groups **(Left)**; cumulative hazard plot of vitiligo in the adenotonsillectomy and non-adenotonsillectomy groups **(Right)**. ATD, adenotonsillar disease.

Figure 2 shows that the adjusted HR for vitiligo in the ATD group was 1.16 (95% CI, 1.09–1.24), indicating that the incidence rate of vitiligo in the ATD group was 1.16 times higher than that in the control group. As a result of subgroup analysis, the adjusted HR for vitiligo after tonsillectomy was 0.82 (95% CI, 0.68–0.99), indicating that the incidence rate of vitiligo in the tonsillectomy group was 0.82 times lower than that in the non-tonsillectomy group.

### Incidence of vitiligo based on other variables

**FIGURE 3:**
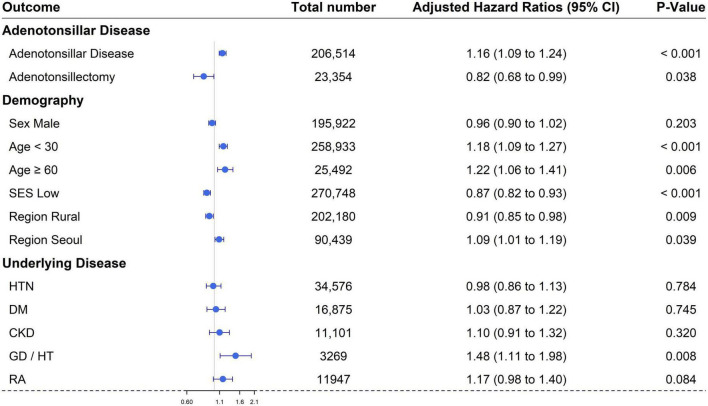
Forest plot of the cumulative hazard ratio of each variable for the development of vitiligo. SES, socio-economic status; HTN, hypertension; DM, diabetes mellitus; CKD, chronic kidney disease; Thyroid, thyroid disease including Graves’ disease and Hashimoto’s thyroiditis; RA, rheumatoid arthritis.

In the comparison between the ATD and non-ATD groups, the relationships between individual variables and vitiligo were as follows: (1) For age, the adjusted HR for the group younger than 30 years was 1.18 (95% CI, 1.09–1.27) and for the group over 60 years was 1.22 (95% CI, 1.06–1.41) compared with the group aged between 30 and 59 years. (2) For sex, the adjusted HR in the male group was 0.96 (95% CI, 0.90–1.02) compared with the female group. (3) The adjusted HRs for residential areas “Seoul,” and “Rural” were 1.09 (95% CI, 1.01–1.19) and 0.91 (95% CI, 0.85–0.98), respectively, compared with the Metropolitan group. (4) In the low economic status group, the adjusted HR was 0.87 (95% CI, 0.82–0.93) compared to the high economic status group. (5–7) For underlying diseases, adjusted HRs for HTN, DM, and CKD groups were 0.98 (95% CI, 0.86–1.13), 1.03 (95% CI, 0.87–1.22), and 1.10 (95% CI, 0.91–1.32), respectively, compared with the equivalent groups without underlying diseases. (8, 9) For comorbid diseases of vitiligo, adjusted HRs for GD/HT and rheumatoid arthritis were 1.48 (95% CI, 1.11–1.98) and 1.17 (95% CI, 0.98–1.40), respectively, compared with the equivalent groups without these comorbid diseases (Table 2 and Figure 3). Among these results, those with the variables of age, residential area, economic status, and GD/HT were statistically significant.

**TABLE 2:** Incidence per 10000 person-years and hazard ratios for vitiligo.

Variable	Total	Cases	10000PY	Hazard ratio	Unadjusted hazard ratio
Total	413,028	4,088			
**ATD**					
Yes	206,514	2,115	9.67	1.16 (1.09–1.24)	1.16 (1.09–1.24)
No	206,514	1,973	8.19	1	1
**Adenotonsillectomy**					
Yes	23,354	209	8.40	0.82 (0.68–0.99)	0.82 (0.68–0.99)
No	23,354	254	10.25	1	1
**Sex**					
Male	195,922	1,906	8.75	0.96 (0.90–1.02)	0.97 (0.91–1.03)
Female	217,106	2,182	9.02	1	1
**Age**					
<30	258,933	2,653	9.21	1.18 (1.09–1.27)	1.15 (1.07–1.23)
30–59	128,603	1,149	8.04	1	1
≥ 60	25,492	286	10.00	1.22 (1.06–1.41)	1.25 (1.10–1.42)
**ES**					
Low	270,748	2,545	8.45	0.87 (0.82–0.93)	0.87 (0.82–0.93)
High	142,280	1543	9.73	1	1
**Region**					
Rural	202,180	1,871	8.30	0.91 (0.85–0.98)	0.92 (0.85–0.99)
Metro	120,409	1,211	9.06	1	1
Seoul	90,439	1,006	10.01	1.09 (1.01–1.19)	1.11 (1.02–1.20)
**HTN**					
Yes	34,576	347	8.94	0.98 (0.86–1.13)	1.01 (0.90–1.12)
No	378,452	3,741	8.89	1	1
**DM**					
Yes	16,875	175	9.26	1.03 (0.87–1.22)	1.04 (0.90–1.21)
No	396,153	3,913	8.88	1	1
**CKD**					
Yes	11,101	122	9.85	1.01 (0.91–1.32)	1.11 (0.93–1.33)
No	401,927	3,966	8.87	1	1
**GD/HT**					
Yes	3,269	48	13.21	1.48 (1.11–1.98)	1.49 (1.12–1.98)
No	409,759	4,040	8.86	1	1
**RA**					
Yes	11,947	138	10.32	1.17 (0.98–1.40)	1.17 (0.99–1.38)
No	401,081	3950	8.85	1	1

ATD, adenotonsillar disease; 10000PY, incidence per 10000-person years; ES, economic status; HTN, hypertension; DM, diabetes mellitus; CKD, chronic kidney disease; GD, Graves’ disease; HT, Hashimoto’s thyroiditis; RA, rheumatoid arthritis; SMD, standardized mean difference.

### Sensitivity analyses

**FIGURE 4:**
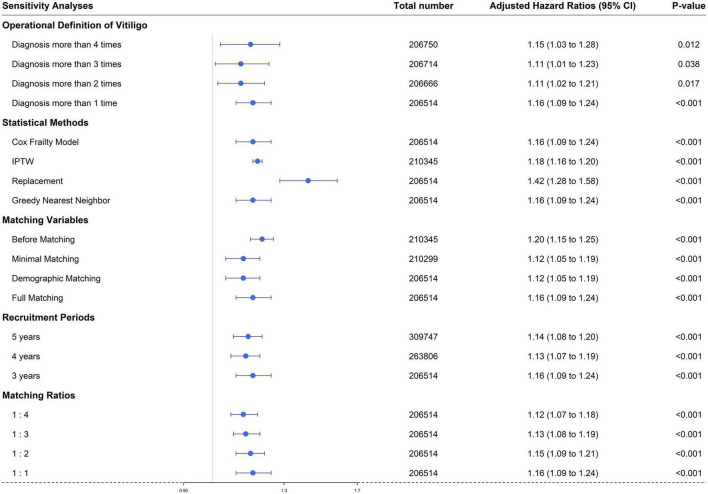
Sensitivity analyses regarding: (1) operational definitions of vitiligo, (2) statistical methods, (3) matching variables, (4) recruitment periods, and (5) matching ratios.

In this test, we could confirm that our result did not change by changing the conditions below (Figure 4). The adjusted hazard ratios for vitiligo, in the ATD group vs. the control group, were as follows: (1) Operational definitions for vitiligo [diagnoses 1 or more times; aHR 1.16 (95% CI, 1.09–1.24), diagnoses 2 or more times; aHR 1.11 (95% CI, 1.02–1.21), diagnoses 3 or more times; aHR 1.11 (95% CI, 1.01–1.23), and diagnoses 4 or more times; aHR 1.15 (95% CI, 1.03–1.28)]. (2) Statistical methods [Cox frailty model; aHR 1.16 (95% CI, 1.09–1.24), IPTW; aHR 1.18 (95% CI, 1.16–1.20), matching with replacement; aHR 1.42 (95% CI, 1.28–1.58), and greedy nearest neighbor; aHR 1.16 (95% CI, 1.09–1.24)]. (3) Matching variables [before matching; aHR 1.20 (95% CI, 1.15–1.25), minimal matching; aHR 1.12 (95% CI, 1.05–1.19), demographic matching; aHR 1.12 (95% CI, 1.05–1.19), and full matching; aHR 1.16 (95% CI, 1.09–1.24)]. (4) Recruitment periods [5 years; aHR 1.14 (95% CI, 1.08–1.20), 4 years; aHR 1.13 (95% CI, 1.07–1.19), and 3 years; aHR 1.16 (95% CI, 1.09–1.24)]. (5) Matching ratios [1:4; aHR 1.12 (95% CI, 1.07–1.18), 1:3; aHR 1.13 (95% CI, 1.08–1.19), 1:2; aHR 1.15 (95% CI, 1.09–1.21), and 1:1; aHR 1.16 (95% CI, 1.09–1.24)].

## Discussion

We performed a nationwide population-based cohort study of 206,514 patients with ATD, and 206,514 controls without ATD, and observed a 1.16-fold higher incidence of vitiligo in the study group with ATD compared with controls with no ATD. Based on these results, we speculate that adenotonsillectomy may attenuate the occurrence of vitiligo. This finding was supported by subgroup analysis of patients with ATD, where the 23,354 patients who underwent adenotonsillectomy had a 0.84-fold lower incidence of vitiligo than the 23,354 controls who did not undergo this surgical procedure.

Until now, the influence of ATD on the development of vitiligo has not been studied. Cho et al. provided the only study investigating the association between adenotonsillectomy and vitiligo; however, their main conclusions were an increased risk of alopecia areata in adenotonsillectomy, and no significant association between vitiligo and adenotonsillectomy (13). The reason for these results, which are inconsistent with our findings, might be because of differences in their study populations and the absence of ATD as a statistical variable. Cho et al. examined a birth cohort who underwent adenotonsillectomy before 9 years of age. They analyzed the association between vitiligo and adenotonsillectomy in those patients over a 17-year study period without considering a diagnosis of ATD as a statistical variable. In its pathogenesis, generalized vitiligo usually has more autoimmune components than segmental vitiligo, and segmental vitiligo is more common in childhood (18). Therefore, patients with segmental vitiligo may be more frequently included in Cho et al.’s birth cohort, which may have biased the results of their study; however, information on the subtype of vitiligo was not available in their study which used the Korean NHIS database. As we conducted our study using cohorts with larger populations of all ages, and which may include all subtypes of vitiligo, we believe our results are more reliable. Furthermore, we used a more representative study design considering the association between vitiligo development and ATD as well as adenotonsillectomy. To our knowledge, this is the first study to identify the risk of vitiligo in patients with ATD. Vitiligo is an autoimmune disorder of the skin where cytotoxic T-cells target and destroy melanocytes resulting in the loss of pigmentation in the skin (19). Elevation of melanocyte antigen-specific CD8 + T-cell levels has been identified in the perilesional skin and blood from vitiligo patients (20). These CD8 + T-cells could infiltrate autologous non-lesional skin explants and efficiently destroy melanocytes (20). Therefore, cytotoxic CD8 + T-cells are both necessary for and capable of obliterating melanocytes in vitiligo skin lesions (19, 21). The various T-cell repertoires, which are fundamental for effective immune function, develop in the thymus. Recent studies have indicated that T-cells can develop extrathymically, although it remains controversial whether extrathymic T-cell development is important for producing the T-cell repertoire in healthy individuals (22). In general, the immunological function of tonsils is very minor compared to that of the thymus, so their function should be compensated by other lymphoid organs in healthy patients who had undergone tonsillectomy. However, McClory et al. presented the possibility that repeated or sustained inflammation gave rise to extrathymic development of T lymphocytes, and that the tonsils may assist in the generation of autoreactive T-cells (22). Similarly, Harabuchi suggested that patients with ATD may have a disrupted immunologic tolerance and their tonsillar mononuclear cells would show a hyper-immune response to bacteria and viruses (10). Then, tonsillar T-cells expressing skin- or kidney-homing receptors are activated and circulated through the bloodstream resulting in palmoplantar pustulosis in the skin or IgA nephropathy in the kidney; these are tonsillar focal diseases (10). From the results of our study, we suggest that recurrent and chronic ATD contributes to the generation of autoreactive T lymphocytes leading to the development of vitiligo, whereas adenotonsillectomy eliminates abnormal T lymphocytes and attenuates the risk of vitiligo development. Moreover, it is assumed that extrinsic insults including toxins or infections initiate autoimmune diseases such as vitiligo (1, 21, 23). Environmental factors such as viral or microbial infections may result in the dysregulation of normal cell processes and induce cellular stress responses which include generation of reactive oxygen species, activation of the unfolded protein response, and initiation of autophagy (21). In vitiligo, these cellular stresses initiate the secretion of signaling molecules from melanocytes whose function is to alert the innate immune system (19). Then, innate immune cells promote the recruitment of adaptive immune CD8 + T-cells to the skin through natural killer cells and dendritic cells (21, 24). Thereafter, CD8 + T-cells detect the abnormal melanocytes and destroy them (19). Hence, in our study, recurrent infections and chronic inflammation from ATD may act as an extrinsic trigger that induces a cellular stress response in melanocytes.

Furthermore, in several studies on the impact of adenoidectomy and/or tonsillectomy on patient immunity, a slight reduction in CD8 + T-cell levels has been observed after surgery (25–27). Although some of these studies showed no statistically significant differences in the postoperative reduction of CD8 + T-cell levels compared with preoperative levels, these data may explain our result, where the postoperative decrease in CD8 + T lymphocytes possibly diminished the development of vitiligo after adenotonsillectomy. Further laboratory investigations are required to evaluate CD8 + T-lymphocyte levels in the blood and perilesional skin of patients with vitiligo, both before and after adenotonsillectomy.

Additionally, we confirmed that thyroid diseases, which include Graves’ disease and Hashimoto’s thyroiditis, is another variable affecting the development of vitiligo, and our results are consistent with those in previous studies. Many studies (7, 28–32) have established an association between vitiligo and thyroid disorders. Bae et al. reported that Graves’ disease and Hashimoto’s thyroiditis were significantly associated with vitiligo in their Korean nationwide population-based study (29). In our study, Graves’ disease and Hashimoto’s thyroiditis were significantly associated with a 1.48-fold higher risk of vitiligo development.

In addition, patients under the age of 30 and over the age of 60 had a significantly increased incidence of vitiligo. In published studies, almost 50% of patients present with vitiligo before 20 years of age, and 70–80% before 30 years of age (33). Howitz et al. reported that the incidence of vitiligo increased with advancing age and its highest prevalence was observed in individuals in the range 60–69 years compared with other age groups (5). One Korean study reported a bimodal peak age distribution in vitiligo patients at 5–15 years and 45–55 years (6). Because these studies and ours reported heterogeneous results, additional investigations are necessary regarding the relationship between patient age and the incidence of vitiligo. Our study found that patients who resided in rural areas and those with low socioeconomic status had decreased risks of vitiligo at 0.87-fold and 0.91-fold, respectively. It is assumed that vitiligo was under-diagnosed in those groups because of difficulties in visiting medical service facilities due to cost and accessibility.

Our study had several limitations. First, we could not consider clinical information from individual patients, including subtypes of vitiligo, extent of vitiligo, age at disease onset, disease duration, and treatment received for vitiligo. Second, as this was an observational study, we could establish an association, but not a causal relationship between ATD or adenotonsillectomy and vitiligo. Third, surveillance bias may have occurred in this retrospective setting. Nevertheless, our study has several strengths. First, its large dataset of 3.5 million individuals from the NIHS database represents real-world data in Korea. Second, propensity score matching imparted further validity to our study, making it equivalent to a randomized controlled trial. Third, inclusion of sensitivity analyses made our results more statistically robust.

## Conclusion

In conclusion, this nationwide population-based cohort study demonstrated the subsequent risk of vitiligo in patients with ATD, possibly through immunological alterations and/or extrinsic triggers of a cellular stress response. Moreover, subgroup analysis indicated that adenotonsillectomy may be a potential preventive measure for vitiligo in patients with ATD, such as TIAS. Our study suggests that clinicians should consider ATD as a pathogenic factor for vitiligo and the potential effect of adenotonsillectomy in its management.

## Data availability statement

The original contributions presented in this study are included in the article/Supplementary material, further inquiries can be directed to the corresponding author.

## Author contributions

JSK and K-HN: conceptualization. MGL, S-WY, M-GK, and JL: data curation and formal analysis. S-KL and I-JL: investigation and visualization. JP and K-HN: methodology. JSK and S-KY: project administration. JP and S-KY: resources. I-JL: software. K-HN: supervision. S-KL and K-HN: validation, writing – original draft preparation, review, and editing. S-KL and I-JL: visualization. All authors contributed to the article and approved the submitted version.
