# Supplementary material for: Association of adenotonsillar disease and adenotonsillectomy with the development of vitiligo: A nationwide population-based cohort study
Source: Front Med (Lausanne). 2022 Nov 3;9:1004218. doi: 10.3389/fmed.2022.1004218 (PMC9669660; doi:10.3389/fmed.2022.1004218)
Supplement: Supplementary file 1 [file Data_Sheet_1.docx]

**Supplementary Table 1. Operational definitions of the variables and outcomes in our study.**

*ATC Code: The main component defined by the first 4 digits of the ATC code.

| ATD | Patients with the ICD codes J35 at least once |
| --- | --- |
| Vitiligo | Patients with the ICD codes L80 at least once |
| HTN | Patients with the following ICD codes who have taken the following ATC code drugs twice or more in 90 days.  ICD-10: I10 I15  ATC code: 1983 1940 1938 1114 1179 1291 4646 4598 4797 4708 1076 4762 4599 4959 2014 6514 6350 6548 6317 6441 6618 6550 6316 5269 6299 6300 6547 6301 5240 5264 6302 5270 6352 6442 5265 6532 5241 6619 5271 6549 6620 5263 6351 6349 6621 6714 6713 6712 6717 6715 6737 4724 4725 5189 6145 4723 6740 6739 6741 5251 5250 5253 6298 5252 6297 |
| DM | Patients with the following ICD codes who have taken the following ATC code drugs twice or more in 90 days.  ICD-10: E10 E11 E12 E13 E14 O24 ATC code : 5259 6396 6453 6273 6361 1753 4413 4849 1701 1704 5074 4618 4887 6268 1915 1654 1320 1658 1656 1657 1006 4062 2490 3480 4319 5011 5008 6133 6242 6164 6191 5121 6266 6445 6397 5273 6282 6743 3795 4302 4861 6538 6557 6539 6540 5188 6319 5237 5236 6728 6727 6738 6725 6720 6718 6721 6726 6719 6647 6420 6499 6648 6501 6485 6486 6500 6418 6646 6419 6484 6267 6667 6670 4434 4435 4743 4972 4986 4719 4742 4211 4529 4527 4981 6303 6304 5256 5255 5023 5247 5029 5137 5022 5070 5196 5071 6305 6306 5185 5186 5207 5205 5206 6357 6356 6321 6398 6414 6320 6541 6450 5238 6490 6495 6494 6492 |
| CKD | Patients with the following ICD codes at least once  ICD-10: N03 N04 N05 N06 N07 N08 N11 N18 N19 N25 N26 N28 N29 |
| GD | Patients with the ICD codes E050 at least once |
| HT | Patients with the ICD codes E063 at least once |
| RA | Patients with the following ICD codes at least once  ICD-10: M05 M06 |
| Adenotonsillectomy | Patients with a corresponding treatment code record between the ATD and Vitiligo diagnoses.  Q2280 Q2281 Q2290 Q2300 Q2310 |

ATD: adenotonsillar disease, HTN: hypertension, DM: diabetic mellitus, CKD: chronic kidney disease, ATD: adenotonsillar disease, GD: Graves’ disease, HT: Hashimoto’s thyroiditis, RA: rheumatoid arthritis

Supplementary Table 2. Characteristics of adenotonsillectomy and non-adenotonsillectomy populations in subgroup analysis

| Variable | Adenotonsillectomy group (Study group, n= 23,354) | Non-Adenotonsillectomy group  (Control group, n= 23,354) | | SMD |
| --- | --- | --- | --- | --- |
| Sex |  |  | 0.00 | |
| Male | 14,046 | 14,039 |  | |
| Female | 9,308 | 9,315 |  | |
| Age |  |  | 0.01 | |
| < 30 | 20,181 | 20,259 |  | |
| 30 ~ 60 | 3,053 | 2,970 |  | |
| $\boldsymbol{\geq}$60 | 120 | 125 |  | |
| ES |  |  | 0.00 | |
| Low | 15,444 | 15,406 |  | |
| High | 7,910 | 7,948 |  | |
| Region |  |  | 0.02 | |
| Rural | 12,784 | 12,608 |  | |
| Metro | 5,942 | 6,065 |  | |
| Seoul | 4,628 | 4,681 |  | |
| HTN |  |  | 0.05 | |
| Yes | 642 | 477 |  | |
| No | 22,712 | 22,877 |  | |
| DM |  |  | 0.00 | |
| Yes | 295 | 290 |  | |
| No | 23,059 | 23,064 |  | |
| CKD |  |  | 0.03 | |
| Yes | 450 | 361 |  | |
| No | 22,904 | 22,993 |  | |
| Thyroid |  |  | 0.02 | |
| Yes | 116 | 92 |  | |
| No | 23,238 | 23,262 |  | |
| RA |  |  | 0.02 | |
| Yes | 324 | 275 |  | |
| No | 23,030 | 23,079 |  | |

ATD: adenotonsillar disease, ES: economic status, HTN: hypertension, DM: diabetes mellitus, CKD: chronic kidney disease, RA: rheumatoid arthritis, SMD: standardized mean difference.
